# Efficacy and safety of pricking-blood therapy for acute gouty arthritis: A protocol for systematic review and meta-analysis

**DOI:** 10.1097/MD.0000000000023521

**Published:** 2020-12-11

**Authors:** Renliang Li, Ping Zhang, Ziyi Hu, Ying Yi, Lisha Chen, Hengyi Zhang, Hong Li, Wanting Liu, Mengwen Wu

**Affiliations:** a Jiangxi University of Traditional Chinese Medicine; b The Affiliated Hospital of Jiangxi University of Traditional Chinese Medicine, Nanchang, Jiangxi Province, PR China.

**Keywords:** acute gouty arthritis, pricking-blood therapy, protocol, systematic review

## Abstract

**Background:**

Acute gouty arthritis is a joint inflammatory reaction that affects the daily quality of patients. Previous reviews of pricking-blood therapy for acute gouty arthritis have been growing, but a systematic review is not available. This study aimed to systematically investigate the efficacy and safety of pricking-blood therapy in treating acute gout arthritis.

**Methods:**

We will search for relevant literature through Chinese and English databases, with the retrieval deadline being December 2020. Databases include PubMed, Embase, Web of Science, the Cochrane Library, China National Knowledge Infrastructure, the Chongqing VIP Chinese Science and Technology Periodical Database, Wanfang Database, and China Biomedical Literature Database. We will also manually search *Chinese Acupuncture & Moxibustion*, *Acupuncture Research,* Chinese Clinical Trial Register, and unpublished studies or references. According to the inclusion and exclusion criteria, the literature will be screened, and the data are extracted independently by the 2 researchers. The primary outcomes were the total effective rate and Visual Analogue Scale (VAS) score. RevMan 5.3.5. software will be used for statistical analysis. According to the Grades of Recommendation, Assessment, Development, and Evaluation (GRADE), each evidence of outcome quality will be appraised.

**Results:**

This study will provide a comprehensive review of current evidence for a pricking-blood therapy treatment for acute gouty arthritis.

**Conclusion:**

The efficacy and safety of picking-blood therapy in treating acute gout arthritis will be evaluated.

**Unique INPLASY number:**

INPLASY2020100094.

## 1 Introduction

Gouty arthritis (GA) is a disease which causes an acute inflammatory reaction when oversaturated monosodium urate (MSU) is deposited in the joints and periarticular tissues.^[1,2]^ It is usually associated with hyperuricemia caused by purine metabolic disorders and decreased uric acid excretion.^[3]^ Acute gouty arthritis (AGA) is characterized by sudden redness, swelling, heat, and pain in the joint and its surrounding soft tissue, usually lasting for several days or weeks and relieves itself. As the disease progresses, the acute attack of GA will be gradually frequent, and the number of joints involved will gradually increase, which will eventually lead to chronic arthritis and joint deformities, which will significantly affect the quality of life of humans.

The global prevalence of gout ranges from <1% to 6.8%, with an annual incidence of 0.58 to 2.89 per thousand people.^[4]^ In China, gout has become the second-largest metabolic disease, with incidence as high as 1.3%.^[5]^ Hyperuricemia and gout are independent risk factors for chronic kidney disease, hypertension, cardiocerebrovascular disease, and diabetes, and are independent predictors of premature death.^[6–8]^ The burden of gout has increased worldwide.^[9]^

The core mechanism of gout is the formation of uric acid monosodium crystals caused by an increase in urate concentration. Although hyperuricemia exists in almost all patients with gout, this biochemical abnormality is not enough to develop clinically apparent joint disease, that is to say, most hyperuricemia patients do not develop gout.^[10]^ Gene mutations and polymorphisms are the genetic basis for the increase in gout.^[11]^ Immunity and inflammation are involved in the pathogenesis of gout.^[12–14]^

At present, there are many kinds of medicines for the treatment of gout, including colchicine, nonsteroidal anti-inflammatory drugs, adrenocortical hormones, and uric acid, which promote or inhibit uric acid production drugs, which can control the disease. However, the choice and use of these existing drugs are limited, and there are many adverse reactions.^[15,16]^ As a result, gout is not well prevented, diagnosed, and treated. Pricking-blood therapy is a kind of external therapy of traditional Chinese medicine (ET-TCM), a long history. In China, it is often used in the treatment of AGA with damp-heat syndrome. Evidence from randomized controlled trials (RCTs) has shown that pricking-blood therapy is an effective way to treat AGA.^[17,18]^ There is still a lack of evidence-based medicine evidence to support it. In this study, we aimed to evaluate the efficacy and safety of pricking-blood therapy for AGA objectively and provide reliable evidence for the clinical application of pricking-blood therapy in AGA.

## 2 Methods

### 2.1 Protocol and registration

This protocol has been registered on the INPLASY website, and the registration number is INPLASY2020100094 (URL https://inplasy.com/inplasy-2020-10-0094/). This report will be performed by the Preferred Reporting Items for Systematic Review and Meta-Analysis Protocols (PRISMA-P).^[19]^

### 2.2 Literature search

We will search the following databases from the establishment to December 2020: PubMed, Embase, Web of Science, the Cochrane Library, China National Knowledge Infrastructure, the Chongqing VIP Chinese Science and Technology Periodical Database, Wanfang Database, and China Biomedical Literature Database. We will also manually search *Chinese Acupuncture & Moxibustion*, *Acupuncture Research,* Chinese Clinical Trial Register, and unpublished studies or references. PubMed's strategy details are summarized in Table 1, and the Chinese databases will use these items translated by Chinese.

**Table 1:** PubMed search strategy.

Number	Search terms
#1	gout[MeSH]
#2	Gouts[title/abstract]
#3	Arthritis, Gouty[MeSH]
#4	Gouty Arthritis[title/abstract]
#5	Arthritides, Gouty[title/abstract]
#6	Gouty Arthritides[title/abstract]
#7	#1 OR #2 OR #3 OR #4 OR #5 OR #6
#8	pricking blood therapy[title/abstract]
#9	pricking, cupping and blood letting therapy [title/abstract]
#10	blood letting[title/abstract]
#11	bloodletting[title/abstract]
#12	pricking bloodletting[title/abstract]
#13	acupuncture and blood-letting[title/abstract]
#14	pricking[title/abstract]
#15	prick[title/abstract]
#16	acupuncture[title/abstract]
#17	needling[title/abstract]
#18	#8 OR #9 OR #10 OR #11 OR #12 OR #13 OR #14 OR #15 OR #16 OR #17
#19	randomized controlled trial[Publication Type]
#20	randomized[Title/Abstract]
#21	placebo[Title/Abstract]
#22	#19 OR #20 OR #21
#23	#7 AND #18 AND #22

### 2.3 Inclusion criteria

The study is considered qualified when the following criteria are met.

(1) Type of studies: RCTs will be included in this review.
(2) Type of participants: Patients who met the standard for AGA diagnosis will be included.
(3) Types of interventions: The treatment group was treated alone with pricking-blood therapy, or pricking-blood therapy combined with conventional therapy and other adjuvant therapy (Chinese herbal medicine, acupuncture, and moxibustion).
(4) Type of comparators: The control group was treated with conventional therapy or other adjuvant therapy without pricking-blood therapy.
(5) Types of outcome measures: The total effective rate and visual analog scale (VAS) score are the primary outcome indicators of this study. This study's secondary outcome is as follows: inflammatory indicators (such as CRP and ESR), uric acid, and incidence of adverse events.

### 2.4 Exclusion criteria

Studies that met the following criteria will be excluded.

(1) Republished literature;
(2) Research on insufficient data or lack of access to the full text;
(3) Case report, Reviews, Basic research, nonRCT.

### 2.5 Studies selection

**Figure 1:**
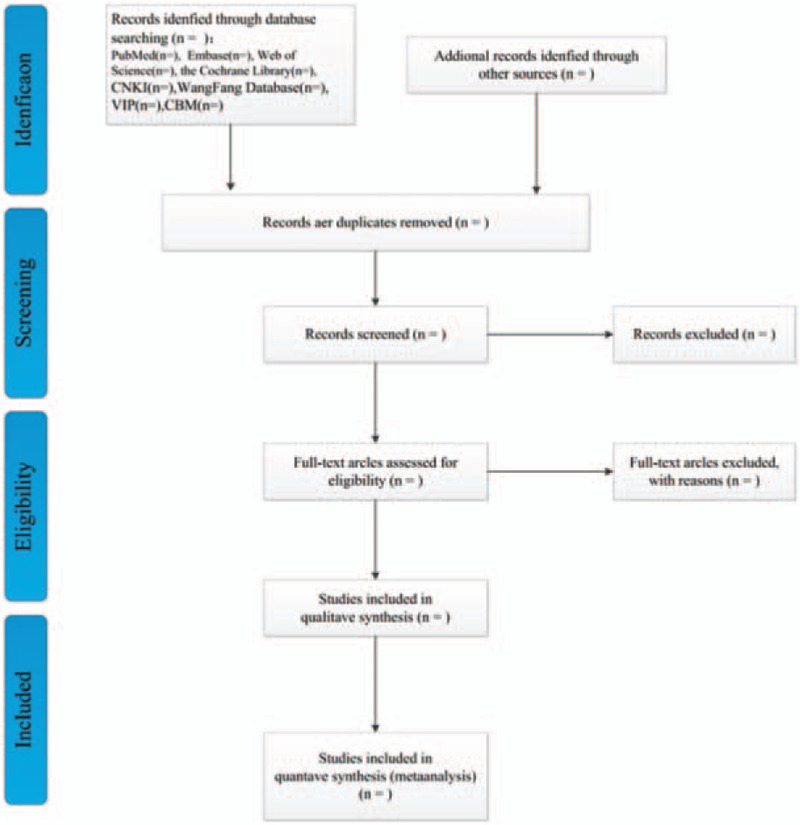
Flow diagram of literature retrieval.

We will eliminate duplicate studies from the search results using Endnote X9 software. Two reviewers will independently screen the literature. Provided that the 2 reviewers have different opinions, whether or not the literature should be included, they should resolve it through discussion. The selection will be performed according to the PRISMA flow chart shown in Figure 1.

### 2.6 Data extraction and management

The data will be screened and extracted by 2 researchers. The content of the data extraction includes the author’ s name, publication year, title, country, enforcement time of the study, study design, sample size, participants, intervention, comparison, outcome, and some relevant characteristics.

### 2.7 Assessment of the methodological quality

There may be biases in clinical trials from selecting and assigning subjects, implementing interventions, following up matters, and measuring and reporting findings at every stage. Thus, RCTs will be evaluated using the bias risk assessment tool (Cochrane Handbook for Systematic Reviews of Interventions).^[20]^ It includes the following 6 items: random sequence generation, allocation concealment, blinding of participants, caregivers, outcome assessors, incomplete outcome data, selective outcome reporting, and other bias. According to each study's results, the 2 researchers made ’low-risk’ ’high-risk’ or ’unclear risk’ assessment of the above 6 items independently. If the 2 researchers have different opinions, the objection will be decided by the third reviewer.

### 2.8 Measures of treatment effect

The risk ratio (RR) and its 95% confidence intervals (95% CIs) will be used as dichotomous variables. Continuous variables were statistically analyzed using weighted mean difference (WMD) or standardized mean difference (SMD) and its 95% CIs.

### 2.9 Dealing with missing data

We will first communicate with the corresponding author to obtain complete data. If we cannot obtain the missing data, we will perform a meta-analysis based on the existing data. If the lost data have no potential impact on our research, we will rule it out.

### 2.10 Assessment of heterogeneity

Heterogeneity was assessed using a Chi-square test and *I*^*2*^ statistics (*P* < .10 or *I*^*2*^ over 50% were defined as substantial heterogeneity).

### 2.11 Data synthesis

RevMan5.3.5 software will conduct this meta-analysis. A random-effects model was used to estimate the pooled primary and secondary outcomes. Forest plots will display the results of the meta-analysis. If the products are not suitable for meta-analysis, we will conduct a descriptive analysis. Only when more than 10 RCTs are included can we use funnel charts to assess publication bias.

### 2.12 Sensitivity analysis

We will use the leave-one-out method for sensitivity analysis to judge the stability of outcome indicators.

### 2.13 Subgroup analysis

If the heterogeneity source cannot be found after sensitivity analysis, we will perform further subgroup analysis.

### 2.14 Summary of evidence

According to the Grades of Recommendation, Assessment, Development, and Evaluation (GRADE), each outcome's evidence quality will be appraised.^[21]^

### 2.15 Ethics and dissemination

Ethical approval is not applicable to this study, as no individual data were available from participants. This systematic review will be published in a peer-reviewed journal.

## 3 Discussion

GA belongs to the “Bi Zheng” category of Traditional Chinese medicine (TCM). This seriously limits the daily activities of patients. In China, damp-heat syndrome is most common in patients with AGA.^[22]^ The theory of TCM holds that pricking-blood therapy can dispel stasis to active blood, clear heat, and drain dampness. It has been reported that pricking-blood therapy may exert its anti-inflammatory effect by upregulating the contents of local anti-inflammatory factors (IL-4 and IL-10).^[23]^ Kai-lu et al^[24]^ also found that regulation of IL-10 gene expression by epigenetic modification of DNA methylation may be one of the crucial mechanisms of the anti-inflammatory effect of pricking-blood therapy. Zheng et al^[25]^ reported that pricking-blood therapy could mediate the TLR4/IL-1 signaling pathway to regulate immune response, reduce inflammatory cell infiltration, and improve local tissue necrosis in patients with GA.

Although RCTs have shown that pricking-blood therapy is safe and effective in gout treatment, it has not been recognized internationally. Consequently, we hope that this study provides a comprehensive review of current evidence for the effectiveness and safety of pricking-blood therapy in AGA treatment and guide clinical decision-making.

## Author contributions

**Conceptualization:** Renliang Li and Ziyi Hu.

**Data curation:** Renliang Li, Ying Yi, and Lisha Chen

**Formal analysis:** Hengyi Zhang, Ping Zhang, and Mengwen Wu

**Funding acquisition:** Ziyi Hu.

**Investigation:** Renliang Li and Ziyi Hu.

**Methodology:** Ping Zhang, Ying Yi, Hong Li, Wanting Liu.

**Resources:** Ziyi Hu.

**Software:** Hong Li, Lisha Chen, and Ping Zhang.

**Supervision:** Renliang Li, Ziyi Hu.

**Writing – original draft:** Renliang Li, Ping Zhang, Ying Yi and Ziyi Hu.

**Writing – review & editing:** Renliang Li, Ping Zhang, and Lisha Chen.
